# Dynamic Bearing–Angle for Vision-Based UAV Target Motion Analysis

**DOI:** 10.3390/s25144396

**Published:** 2025-07-14

**Authors:** Yu Luo, Hongwei Fu, Tingting Fu, Hao Cha, Bing Tian, Huatao Tang, Feng Liu

**Affiliations:** 1 College of Electronic Engineering, Naval University of Engineering, Wuhan 430033, China; d22381001@nue.edu.cn (Y.L.); d22181002@nue.edu.cn (H.F.); futt361@163.com (T.F.); hydchj@126.com (H.C.); sweetybox123@163.com (B.T.); ch20030708@163.com (F.L.); 2 Air Force Early Warning Academy, Wuhan 430033, China

**Keywords:** target motion estimation, robustness enhancement, target detection frame jitter

## Abstract

The Bearing–Angle algorithm effectively improves the observability of vision-based motion estimation for moving targets by combining the dimensional information of target detection frames. However, the robustness of this algorithm will be significantly reduced when the observation error increases due to sudden changes in the target motion state. To address this shortcoming, this paper proposes a visual target motion estimation algorithm called the Dynamic Bearing–Angle, which aims to improve the accuracy and robustness of target motion analysis in dynamic scenarios such as unmanned aerial vehicle (UAV). The algorithm innovatively introduces a dual robustness mechanism of dynamic noise intensity adaptation and outlier suppression based on M-estimation. By adjusting the noise covariance matrix in real time and assigning low weights to the outlier observations using the Huber weight function, the Dynamic Bearing–Angle algorithm is able to effectively cope with non-Gaussian noise and sudden target maneuvers. We validate the performance of the proposed algorithm with numerical simulations and real sensor data, and the results show that the Dynamic Bearing–Angle maintains good robustness and accuracy under different noise intensities.

## 1. Introduction

Vision-based target motion estimation algorithms have gained prominence due to their cost-effectiveness, high information density, real-time responsiveness, and adaptability to dynamic environments. Compared with communication-based methods, vision approaches are inherently immune to electromagnetic interference and require no additional hardware or specialized detection algorithms, making them highly integrable and extensible. These advantages have driven their extensive application in UAV swarms, counter-UAV detection, and drone pursuit missions [1,2,3].

Vision-based motion estimation algorithms rely on bounding boxes derived from visual target detection in pixel coordinates to infer target kinematics. Based on the type of input information, these algorithms are broadly categorized into two classes: Bearing-Only [4] and Bearing–Angle [5,6]. The Bearing-Only method, the earliest and most widely used approach, estimates target position and velocity by combining the pixel coordinates of bounding box centers (obtained from detection algorithms) with a pinhole camera model to compute spatial bearing vectors [7,8].

The Bearing-Only algorithm suffers from significant limitations. This approach exhibits high sensitivity to variations in the physical dimensions of targets under different viewing angles. When rapid or substantial changes in the target size occur, the algorithm’s accuracy and robustness degrade considerably [9,10]. To address this issue, the observer must perform lateral motion orthogonal to the target’s bearing vector, providing additional perspectives to enhance observability. Aidala et al. [11] theoretically demonstrated that velocity estimation remains unbiased under such conditions, while position bias can be reduced through optimized observer trajectories. Consequently, researchers have explored controlled observer motion patterns to improve observability in three-dimensional space [12,13,14]. However, these observer maneuvers often conflict with mission-specific motion requirements. Building upon Bearing-Only research, Ning et al. proposed the Bearing–Angle algorithm [5]. By fusing bearing vectors with angular span measurements, the Bearing–Angle fully utilizes previously underexploited bounding box dimension information, significantly enhancing observability. This innovation eliminates dependence on high-agility lateral maneuvers, permitting direct approach trajectories toward targets—a feature particularly advantageous for downstream control tasks. Furthermore, the Bearing–Angle employs Pseudo-Linear Kalman Filtering (PLKF) [15], which avoids complex Jacobian computations in traditional nonlinear filtering through state reparameterization, thereby improving numerical stability.

**Figure 1:**
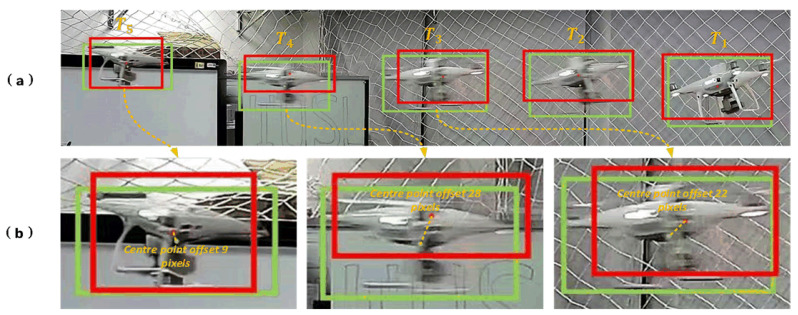
Schematic diagram of visual localization error analysis. (**a**) Detection effects during typical phases of UAV continuous motion: The UAV performs a horizontal “acceleration-constant velocity-deceleration” maneuver. Phases T1– T3 exhibit significant rightward acceleration, while phases T3–T5 transition to deceleration with sustained rightward motion. Detection results are visualized through dual markers: Red bounding boxes represent real-time outputs from a well-trained YOLOv5 model [18], and green ground-truth boxes denote professionally annotated references. Their centers are marked by red and green dots, respectively, where spatial offsets directly reflect localization errors. (**b**) Spatiotemporal comparative analysis of the T3–T5 deceleration phase: Jitter in bounding boxes caused by motion blur and sensor noise is quantified. Smaller jitter occurs at slower UAV speeds, while rapid motion induces severe jitter, with pixel offsets of bounding box centers reaching 20–30 pixels.

**Figure 2:**
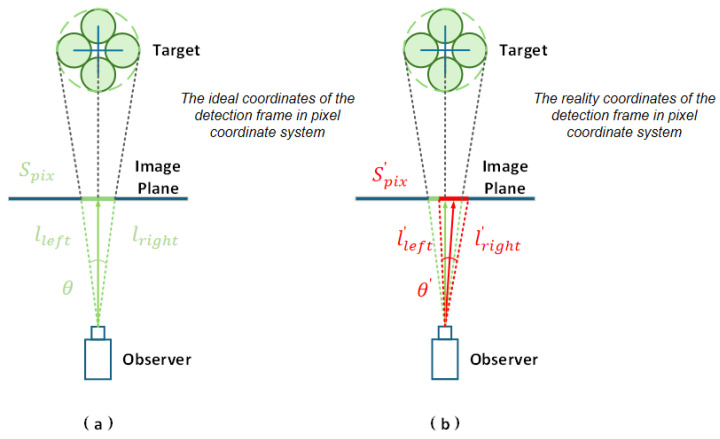
Bounding frame without noise influence and without jitter in the pixel coordinate system under ideal conditions (as shown in (**a**) in the figure). In reality, the bounding box is disturbed by noise, and the left and right boundary positions of the bounding box, the position of the center point, and the size of the boundary fluctuate (as shown in (**b**) in figure), which in turn affects the observed values of Bearing and Angle.

However, our field deployment reveals critical limitations of traditional Bearing-Only and its derivative Bearing–Angle approaches in real-world UAV motion scenarios. These methods exhibit excessive dependence on the accuracy of bounding boxes provided by upstream detection trackers, demonstrating high sensitivity to detection noise [16,17]. Unfortunately, during abrupt UAV maneuvers, the bounding box precision from detection algorithms suffers rapid fluctuations, causing significant jitter with varying amplitudes (Figure 1). This phenomenon severely degrades the measurement accuracy of both the Bearing–Angle and the angular spans (Figure 2). Moreover, the frequent and nonlinear nature of bounding box jitter introduces substantial noise uncertainty throughout the estimation pipeline.

Under such circumstances, the assumption of fixed noise covariance contradicts the time-varying nature of noise statistical characteristics in real-world scenarios, rendering this framework incapable of adapting to sudden target maneuvers or intense external interference. Conventional filtering algorithms fundamentally fail to address the degradation in state estimation performance caused by non-Gaussian noise arising from abrupt changes in UAV motion states. These limitations highlight the imperative need to develop adaptive nonlinear filtering architectures capable of coordinating observation uncertainty with dynamic environmental constraints.

Therefore, to effectively manage and suppress these noise sources while enhancing algorithmic robustness and accuracy, we propose a novel Dynamic Bearing–Angle motion estimation algorithm. This algorithm establishes a dual robust mechanism integrating “outlier suppression via weighting” and “dynamic noise intensity adaptation” through the fusion of M-estimation into dynamic filtering: (1) The Huber weight function introduced via M-estimation assigns low weights to abnormal observations such as sudden outliers caused by transient intense jitter in detection bounding boxes, significantly enhancing robustness against non-Gaussian noise including impulsive and heavy-tailed noise. (2) Concurrently, the real-time adaptive adjustment of noise covariance matrices through dynamic filtering precisely tracks variations in noise statistics, accelerates adaptation to system dynamic changes such as target maneuvers, and reduces convergence time.

In summary, our principal contributions are threefold:

- We propose a dynamic filtering mechanism based on real-time noise statistics with dynamic smoothing factor adjustment. This addresses the parameter mismatch issue of traditional fixed covariance models in non-stationary noise scenarios, thereby improving estimation accuracy and convergence speed.
- We innovatively integrate M-estimation with filtering algorithms by dynamically allocating observation weights through the Huber robust loss function. This approach assigns low weights to abnormal data caused by sudden jitter in detection bounding boxes or sensor outliers, overcoming the dependency of traditional filtering on Gaussian noise assumptions. The enhanced robustness effectively prevents outliers from dominating state estimation.
- A Dynamic Kalman Filter framework is constructed based on a dual robust mechanism of weights to suppress outliers and dynamic adaptation of noise intensity in parallel. It suppresses observational anomalies through the anti-differential property of M-estimation and compensates model uncertainty through the adaptive property of dynamic filtering. The filtering framework can handle more complex noise types and significantly extends the generalizability of the algorithm in high-noise and strong dynamic scenarios.

## 2. Related Works

Vision-based target motion estimation methods like Bearing-Only and Bearing–Angle face limitations in noise robustness and observability. This section systematically reviews the theoretical frameworks and practical challenges of existing techniques from two dimensions: vision-based estimation algorithms and adaptive filtering methods, providing a theoretical reference for the proposed Dynamic Bearing–Angle algorithm.

### 2.1. Visual-Based Target Motion Estimation

In the field of vision-based target motion estimation, Bearing-Only and Bearing–Angle methods provide critical theoretical frameworks for position and velocity estimation by measuring target azimuth angles and angular spans through monocular or binocular vision. However, their practical implementation faces multiple challenges. The noise amplification characteristics of nonlinear systems significantly magnify minor visual measurement errors during geometric inversion. Bearing-Only methods rely on strongly coupled nonlinear relationships between azimuth vectors and observer positions, causing the extended Kalman filter (EKF) to accumulate linearization errors during abrupt target maneuvers, which may lead to algorithm divergence [7,19,20]. While Bearing–Angle methods improve observability by incorporating angular measurements, the nonlinear correlation between target physical dimensions and distances further complicates state estimation, imposing stricter demands on noise suppression capabilities [21]. Observability constraints also limit application scenarios: Bearing-Only methods require high-order observer maneuvers (e.g., continuous lateral motion) to satisfy observability conditions [8], which conflicts with mission objectives of platforms like UAVs and risks observability matrix degradation under non-ideal observer motions in 3D dynamic environments. Although Bearing–Angle methods reduce maneuver dependency, target size sensitivity introduces new uncertainties, where dimensional estimation errors propagate nonlinearly to directly degrade distance accuracy.

Regarding the trade-off between computational efficiency and estimation precision, existing methods are constrained by algorithm complexity and real-time requirements. Pseudo-Linear Kalman Filters (PLKFs) achieve computational efficiency through linear approximations [5,9], but their neglect of non-Gaussian noise and measurement matrix correlations induces progressive biases. Particle filters can address strongly nonlinear problems [22], yet their computational complexity struggles to meet real-time demands in scenarios like UAV obstacle avoidance. Three-dimensional extension schemes (e.g., spherical coordinate models [7]) enhance spatial adaptability but introduce additional state uncertainties due to latitude angle singularities. Furthermore, robustness limitations hinder method generalization: Bearing-Only methods easily lose observability under constant-velocity linear target motions [23], while Bearing–Angle methods exhibit sensitivity to time-varying target dimensions, necessitating online parameter identification mechanisms. Neither approach sufficiently models non-Gaussian noise characteristics in dynamic environments, resulting in systematic biases for Gaussian-assumption-based filters in complex scenarios [24]. These challenges collectively highlight the core bottleneck of current vision-based motion estimation technologies: achieving balanced optimization among noise suppression, real-time performance, and environmental adaptability remains a critical unresolved issue.

### 2.2. Adaptive Filtering Algorithm

In the field of visual target motion estimation, adaptive filtering techniques have emerged as core solutions to address challenges including nonlinear noise amplification, observability constraints, and real-time requirements through dynamic adjustment of noise models and state estimation strategies.

Within adaptive filtering, three representative methods—Sage–Husa [25], Adaptive Particle Filter (APF) [26], and Adaptive Unscented Kalman Filter (AUKF) [27]—demonstrate distinct advantages and limitations. The Sage–Husa algorithm significantly enhances sensor data accuracy and reliability through real-time estimation of system and measurement noise statistics. Its adaptive parameter adjustment mechanism dynamically optimizes Kalman gain and covariance matrices, improving robustness in nonlinear and non-Gaussian systems while maintaining computational efficiency approximately 10% lower than traditional Kalman filters. However, this method exhibits higher implementation complexity requiring meticulous tuning of multiple parameters and demonstrates sensitivity to initial noise statistics where improper initialization may degrade filtering performance. In contrast, the Adaptive Particle Filter (APF) excels in harmonic suppression and reactive power compensation scenarios through dynamic compensation capabilities, effectively eliminating multiple/high-order harmonics while avoiding resonance issues. Nevertheless, its high hardware costs and limited filtering capacity restrict applications in large-scale power grids or high-voltage environments. For nonlinear system state estimation, the Adaptive Unscented Kalman Filter (AUKF) preserves data distribution characteristics via unscented transformation and integrates adaptive noise adjustment mechanisms, achieving high-precision state estimation in complex scenarios such as autonomous vehicle perception and control. Despite superior nonlinear processing capabilities and adaptability, AUKF’s mathematical complexity and computational demands pose challenges for real-time critical systems like high-frequency visual tracking.

Additionally, the adaptive Kalman filtering algorithm proposed by A. H. MOHAMED and K. P. SCHWARZ in 1999 [28] has exerted significant influence in the field of INS/GPS integrated navigation. This algorithm significantly enhances the performance and robustness of Kalman filtering in complex environments through adaptive adjustment of noise covariance matrices. Similarly, the adaptive covariance-tuning Kalman filtering method developed by AKHLAGHI et al. [29] in 2017 has demonstrated excellent performance in dynamic state estimation for power systems. While these methods share conceptual similarities with our research, our dynamic Kalman filtering algorithm exhibits unique innovations in three critical aspects: application scenarios, dynamic adjustment mechanisms, and system modeling approaches.

## 3. Method

To address the robustness issues in dynamic noise scenarios, this chapter presents the Dynamic Bearing–Angle algorithm, establishing a dual robust mechanism of “dynamic noise intensity adaptation-outlier suppression”. It introduces the data processing flow, system modeling, and estimator design, leveraging the Huber weight function and adaptive covariance adjustment to enhance anti-interference capabilities.

### 3.1. Dynamic Bearing–Angle Overview

**Figure 3:**
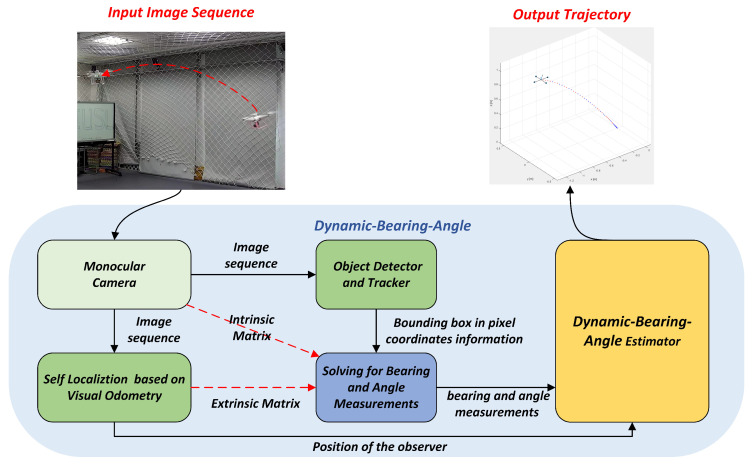
Overview of Dynamic Bearing–Angle. The Dynamic Bearing–Angle algorithm firstly captures the target image sequence by a monocular camera, extracts the target bounding box information by using the 2D target detector and tracker, and at the same time parses the VIO self-localization information according to the image sequence acquired by the observer. Then, the azimuth and angle measurements of the target are calculated by combining the internal and external reference matrices of the camera, the VIO self-localization information of the observer, and the target bounding box information. Finally, the measured values are input into the target motion estimator to predict the target’s motion trajectory information.

The architecture in Figure 3 clearly shows the entire flow of the Dynamic Bearing–Angle algorithm from data acquisition to state estimation and how these steps are integrated into a unified framework. It acquires image data of the target using a monocular camera. After capturing the image data of the target, it is fed into the 2D target detector to predict the bounding box information (center point coordinates and size information) of the target. Subsequently, the inner reference matrix, outer reference matrix, and bounding box information of the camera are combined to solve the Bearing and Angle measurements in the world coordinate system. Finally, these measurements, combined with the self-localization information obtained by the observer using the VIO, are injected into the target motion estimator to estimate the motion state of the target.

### 3.2. System Modeling

The motion state matrix and observation matrix are modeled for the UAV motion estimation nonlinear system problem. The target motion state equation can be expressed as

(1) $$x_{k+1}=Fx_{k}+Q_{K}.$$

where $x_{k}={\left[P_{T},V_{T},\ell \right]}^{T}\in R^{7}$ is the state vector at the time *k*, containing the target’s position $P_{T}$, velocity $V_{T}$, and physical dimensions *ℓ* (one-dimensional physical linear dimensions). $Q_{k}$ is the process noise that satisfies zero-mean Gaussian white noise, and *Q* is the covariance matrix of the process noise that satisfies $Q=diag\left(0,0,0,\sigma_{v}^{2},\sigma_{v}^{2},\sigma_{v}^{2},\sigma_{\ell }^{2}\right)\in R^{7\times 7}$. *F* is the state transfer function, and, in combination with the sampling time δt, it can be expressed as

(2) $$F_{x_{k}}=\left[\begin{array}{ccc} I_{3\times 3} & \delta tI_{3\times 3} & 0_{3\times 1}\\ 0_{3\times 3} & I_{3\times 3} & 0_{3\times 1}\\ 0_{1\times 3} & 0_{1\times 3} & 1 \end{array}\right]x_{k}\in R^{7\times 7}.$$

The exclusion of direct acceleration modeling in this framework stems from the observation that the motion of targets can be approximated as uniform linear motion in most scenarios, particularly over short time intervals. This assumption enables model simplification by allowing the state transition function F to be described through a dual-integration model. The dual-integration model postulates linear temporal evolution of both velocity and position parameters, which provides sufficient approximation for numerous practical applications. Moreover, acceleration parameters are generally not directly retrievable through visual measurement systems. Even when acceleration components exist, their influence on visual measurements may prove negligible or unstable, especially during brief observation periods. Thus, the inherent unobservability of acceleration components could introduce additional uncertainties and measurement noise if forcibly incorporated into the state transition function.

According to the corresponding physical model [13], we can obtain the mathematical expressions of the measurement equations corresponding to the bearing vector *g* and the tension angle θ of the target:

(3) $$g=\frac{R_{c}^{w}P_{cam}^{-1}q_{pix}}{∥R_{c}^{w}P_{cam}^{-1}q_{pix}∥}=\frac{P_{T}-P_{O}}{r},$$

(4) $$\theta =arccos\frac{l_{left}^{2}+l_{right}^{2}-s_{pix}^{2}}{2l_{left}^{2}l_{right}^{2}}=2arctan\left(\frac{\ell }{2r}\right)\approx \frac{\ell }{2r}.$$

For the vectors *g*, $P_{cam}\in R^{3\times 3}$ is the internal reference matrix of the camera, $R_{c}^{w}$ is the rotation matrix from the camera frame to the world frame; $q_{pix}={\left[x_{pix},y_{pix},1\right]}^{T}\in R^{3}$; $\left(x_{pix},y_{pix}\right)$ is the center coordinate under the pixel coordinate system of the detection frame; $P_{O}$ is the position of the observer, $P_{T}$ is the position of the target, and $r=∥P_{T}-P_{O}∥$ is the distance between the target and the observer. Then, for θ, $l_{left}$ and $l_{right}$ are the pixel distances from the center of the camera to the left and right midpoints of the bounding box, respectively, and $s_{pix}$ denotes the size of the pixel of the bounding box.

After obtaining the measurements, we relate the target state to the measurements using the measurement equation, which is expressed as

(5) $$z_{k}=h\left(x_{k}\right)+R_{k}.$$

where $z_{k}$ is the observation vector. $h(x_{k})$ is a nonlinear observation model that maps the state vector $x_{k}$ to the observation space. $R_{k}$ is the observation noise vector, which represents the uncertainty in the observation process and is usually assumed to be zero-mean Gaussian white noise.

The covariance matrix of the observation noise *R* is the covariance matrix of the observation noise $R_{k}$. If the measurement noise of bearing vector *g* and angle θ are independent and have known noise variances $\sigma_{\mu }^{2}$ and $\sigma_{w}^{2}$, respectively,

(6) $$R=\left[\begin{array}{cc} \sigma_{\mu }^{2}I_{3\times 3} & 0_{3\times 1}\\ 0_{1\times 3} & \sigma_{w}^{2} \end{array}\right]$$

Specifically, the observation model $h(x_{k})$ can be written as

(7) $$h\left(x_{k}\right)=\left[\begin{array}{c} h_{g}\left(P_{T},P_{O}\right)\\ h_{\theta }\left(P_{T},P_{O},\ell \right) \end{array}\right]=\left[\begin{array}{c} \frac{P_{T}-P_{O}}{∥P_{T}-P_{O}∥}\\ 2arctan(\frac{\ell }{2∥P_{T}-P_{O}∥}) \end{array}\right]$$

In order to find the Jacobian matrix $H_{k}={\left[H_{g},H_{\theta }\right]}^{T}$ for the observation equation, it is necessary to find the Jacobian matrix $H_{g}$ for the bearing vector *g* and the Jacobian matrix $H_{\theta }$ for the angle θ, respectively:

(8) $$H_{g}=\frac{\partial h_{g}\left(x_{k}\right)}{\partial x_{k}}=\left[\begin{array}{ccccccc} \frac{\partial g_{x}}{\partial T_{x}} & \frac{\partial g_{x}}{\partial T_{y}} & \frac{\partial g_{x}}{\partial T_{z}} & 0 & 0 & 0 & 0\\ \frac{\partial g_{y}}{\partial T_{x}} & \frac{\partial g_{y}}{\partial T_{y}} & \frac{\partial g_{y}}{\partial T_{z}} & 0 & 0 & 0 & 0\\ \frac{\partial g_{z}}{\partial T_{x}} & \frac{\partial g_{z}}{\partial T_{y}} & \frac{\partial g_{z}}{\partial T_{z}} & 0 & 0 & 0 & 0 \end{array}\right]$$

(9) $$H_{g}=\frac{\partial h_{\theta }\left(x_{k}\right)}{\partial x_{k}}=\left[\begin{array}{ccccccc} \frac{\partial \theta }{\partial P_{T_{x}}} & \frac{\partial \theta }{\partial P_{T_{y}}} & \frac{\partial \theta }{\partial P_{T_{z}}} & 0 & 0 & 0 & \frac{\partial \theta }{\partial \ell } \end{array}\right]$$

where $T_{x}$, $T_{y}$, and $T_{z}$ represent the components of the target’s position vector $P_{T}$ in the world coordinate system.

Solving Equations (8) and (9) jointly and combining the known conditions $r=∥P_{T}-P_{O}∥$, the complete $H_{k}$ is expressed as

(10) $$H_{g}=\left[\begin{array}{ccccccc} \frac{\partial g_{x}}{\partial T_{x}} & \frac{\partial g_{x}}{\partial T_{y}} & \frac{\partial g_{x}}{\partial T_{z}} & 0 & 0 & 0 & 0\\ \frac{\partial g_{y}}{\partial T_{x}} & \frac{\partial g_{y}}{\partial T_{y}} & \frac{\partial g_{y}}{\partial T_{z}} & 0 & 0 & 0 & 0\\ \frac{\partial g_{z}}{\partial T_{x}} & \frac{\partial g_{z}}{\partial T_{y}} & \frac{\partial g_{z}}{\partial T_{z}} & 0 & 0 & 0 & 0\\ \frac{\partial g_{z}}{\partial T_{x}} & \frac{\partial g_{z}}{\partial T_{y}} & \frac{\partial g_{z}}{\partial T_{z}} & 0 & 0 & 0 & 0 \end{array}\right]$$

### 3.3. Dynamic Bearing–Angle Estimator

#### 3.3.1. Estimator Construction

**Figure 4:**
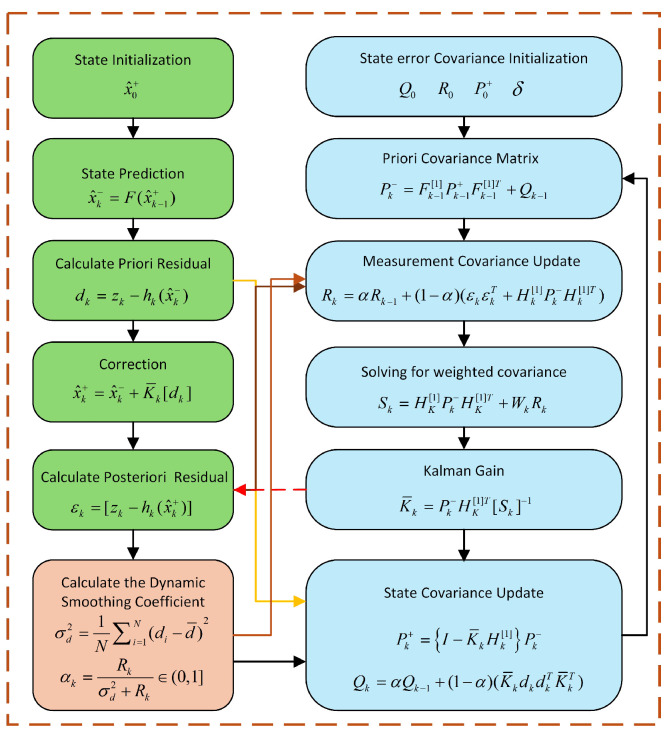
Flowchart of Dynamic Bearing–Angle Estimator.

After establishing the state transition equations and measurement equations through modeling, target motion estimation can be achieved using filtering algorithms. However, conventional filtering algorithms fail to adequately address the dynamic noise issues inherent in UAV target motion estimation tasks. To overcome this limitation, this paper proposes a novel Dynamic Kalman Filter and constructs a Dynamic Bearing–Angle Estimator accordingly. The comprehensive workflow is illustrated in Figure 4. The proposed algorithm comprises four distinct phases: Initialization, Time Update, Measurement Update, and Dynamic Adjustment.

1. Initialization step: In the initialization step, we need to establish the initial process noise covariance matrix $Q_{0}$ and measurement noise covariance matrix $R_{0}$, as well as the initial state estimate ${\hat{x}}_{0}^{+}$ and state covariance matrix $P_{0}^{+}$. Here, “+” indicates that the estimate is a posteriori. Also, we initialize the M-estimation parameter δ.
2. Time Update step: We then perform Time Update; at which point, we need to predict the state and predict the state covariance matrix separately:
  (11) $${\hat{x}}_{k}^{-}=F\left({\hat{x}}_{k-1}^{+}\right),$$
  (12) $$P_{k}^{-}=F_{k-1}^{\left[1\right]}P_{k-1}^{+}F_{k-1}^{[1]T}+Q_{k-1}.$$
  Here, “−” indicates that the estimate is a priori. $F_{k-1}^{\left[1\right]}$ denotes the state transfer function at time step k−1, and “[1]” in the equation is to distinguish between different time steps.
3. Measurement Update step: We need to calculate the residuals first:
  (13) $$d_{k}=z_{k}-h_{k}\left({\hat{x}}_{k}^{-}\right),$$
  (14) $$\varepsilon_{k}=\left[z_{k}-h_{k}\left({\hat{x}}_{k}^{+}\right)\right],$$
  It is important to note that $d_{k}$ is the difference between actual measurements and predicted measurements based on a priori estimates, $d_{k}={\left[d_{g}^{T},d_{\theta }\right]}^{T}$; $\varepsilon_{k}$ is the difference between actual measurements and predicted measurements based on a posteriori estimates.
  Then, we introduce outlier suppression based on M-estimation by constructing a weight matrix $W_{k}\in R^{4\times 4}$ using the Huber loss function to dynamically weight the residual components:
  (15) $$w_{k,i}=\left\{\begin{array}{cc} 1 & \mathrm{if}\;\left|\frac{d_{k,i}}{\delta_{k,i}}\right|\leq \delta \\ \frac{\delta_{k,i}}{\left|\frac{d_{k,i}}{\delta_{k,i}}\right|} & \mathrm{if}\;\left|\frac{d_{k,i}}{\delta_{k,i}}\right|>\delta \end{array}\right.$$
  where $\sigma_{k,i}$ is the standard deviation of the observation noise, and the effect of outliers is suppressed by a threshold δ. The threshold parameter is set to $\delta =1.345\sqrt{tr(R_{k})/4}$, where $R_{k}$ is the observation noise covariance matrix, and $tr(R_{k})/4$ denotes the average noise variance of the observation vector. This setup ensures that δ matches the expected level of the observation noise without the need for manual parameterization.
  The filtering update using the weighting matrix and solving for the weighted posterior residual covariance after calculating the computational Jacobi matrix obtain
  (16) $$S_{k}=H_{k-1}^{\left[1\right]}P_{k-1}^{-}H_{k-1}^{[1]T}+W_{k}R_{k}W_{k},$$
  Update the Kalman gain:
  (17) $${\bar{K}}_{k}=P_{k}^{-}H_{k}^{[1]T}{\left[S_{k}\right]}^{-1},$$
  $H_{k}^{\left[1\right]}$ denotes the Jacobi matrix of the measurement function at time step k−1.
  The a posteriori state estimates and covariances are finally obtained:
  (18) $${\hat{x}}_{k}^{+}={\hat{x}}_{k}^{-}+{\bar{K}}_{k}\left[d_{k}\right],$$
  (19) $$P_{k}^{+}=\left\{I-{\bar{K}}_{k}H_{k}^{\left[1\right]}\right\}P_{k}^{-}.$$
4. Dynamic Adjustment step: At this time, the system needs to manually adjust the process noise and measurement noise covariance matrices relying on experience and trial and error. In reality, however, the process noise and measurement noise of the system are not fixed, especially in the dynamic situation that the UAV is in, where the noise characteristics may change over time. In this condition, manual adjustment of the covariance matrix may not be able to accommodate these changes in a timely manner. We propose to optimize the performance of the filter by dynamically adjusting the covariance matrix using the errors in the analytical prediction and update steps in order to overcome the problems posed by the system in a dynamic noise environment.
  For the process noise covariance matrix *Q*, we use the difference between the actual measurements and the predicted measurements based on a priori state estimation to dynamically adjust, which ensures that the system can accurately change dynamically. For the measurement noise covariance matrix *R*, we adjusted it using the difference between the actual measurements and the predicted measurements based on a posteriori state estimation to ensure that the filter accurately modeled the measurement error.
  These two approaches work together to enable the original Kalman filter to automatically adapt to changes in the noise characteristics of the system and improve the accuracy and robustness of the state estimation. The detailed calculation formula is as follows:
  (20) $$Q_{k}=\alpha Q_{k-1}+\left(1-\alpha \right)\left({\bar{K}}_{k}d_{k}d_{k}^{T}{\bar{K}}_{k}^{T}\right),$$
  (21) $$R_{k}=\alpha R_{k-1}+\left(1-\alpha \right)\left(\varepsilon_{k}\varepsilon_{k}^{T}+H_{k}^{\left[1\right]}P_{k}^{-}H_{k}^{[1]T}\right).$$
  Also, considering that the system is in a dynamically changing environment, the extreme noise characteristics may cause the system sensitivity to be too high when a fixed value of α may lead to unstable filter performance. Therefore, we include a dynamic adjustment rule for the smoothing coefficient α in adjusting the covariance matrix, which dynamically adjusts its value according to the magnitude of real-time data or noise.
  We use the variance adjustment of the prior residual series. To illustrate, a larger-than-expected variance in the prior residual series may indicate an underestimation of measurement noise, which can then be reduced. Specifically, the sample variance of the prior residual series is first calculated as a measure of prior residual intensity:
  (22) $$\sigma_{d}^{2}=\frac{1}{N}\sum_{i=1}^{N}{\left(d_{i}-\bar{d}\right)}^{2}.$$
  Based on the relations between the variance of the prior residual sequence and the measurement noise covariance, we designed the following formula for adaptive adjustment:
  (23) $$\alpha_{k}=\frac{R_{k}}{\sigma_{d}^{2}+R_{k}}\in \left(0,1\right].$$
  For systems with suboptimal initial conditions or inaccurate models, dynamic tuning can help the filter converge to the correct state estimate more quickly. Adaptive tuning can improve the robustness of the filter in the face of external disturbances or internal system uncertainties. This adaptive setting approach can further improve the performance of the filter, especially when the noise intensity of the system changes rapidly.

#### 3.3.2. Estimator Coupling Relationship Analysis

The DEKF and M-estimation fusion mechanism proposed in this paper does not work independently but creates a synergistic effect through the closed-loop coupling of cross-level noise suppression and covariance adaptation. Two aspects are developed below.

1. Hierarchical Complementation of Noise Suppression and Statistical Adaptation:
  The M-estimation weighs the discrete outliers generated by the sudden jitter of the detection frame through the Huber weight function to construct an anomaly filter barrier in the observation layer, while the DEKF tracks the continuously time-varying characteristics of sensor noise through real-time updating of the noise covariance matrices *Q* and *R*. The M-estimation is based on a combination of the following two methods. When the jitter of the detection frame generates sudden noise, the estimation M first reduces the weight of the noise so that the residual sequence of the DEKF covariance calculation is closer to the true distribution; DEKF then adjusts *Q* and *R* based on the `’purified” residuals to form a cascading effect of `’suppression followed by adaptation”. The DEKF then adjusts *Q* and *R* according to the `’purified” residuals, forming a cascade effect `’suppression and adaptation”.
2. Lost Loop for Error Compensation in Nonlinear Systems:
  In the target maneuvering scenario, DEKF compensates for the uncertainty of the state transfer model by adjusting the Q matrix, while M-estimation mitigates the error amplification caused by the nonlinearity of the measurement model through weight assignment. The two form a mathematical coupling through the residual sequence: the weight matrix generated by M-estimation reconstructs the observation noise covariance, while the covariance updating formula of DEKF relies on the statistical properties of the weighted residuals, which ultimately results in a positive iterative cycle of “outlier suppression → covariance optimization → state estimation accuracy enhancement”.

## 4. Numerical Simulation Experiments

To validate the algorithm performance, three simulation scenarios (target stationary, linear motion, and cooperative maneuvering) are designed. By comparing with baselines like Bearing-Only, the estimation accuracy and convergence speed under different noise intensities are analyzed, verifying the algorithm’s effectiveness in dynamic environments.

### 4.1. Simulation Experiment Setup

- Implement details

The hardware device is Intel (R) Core (TM) i7-10700F CPU @ 2.90 GHz with 16 GB of installed memory, Windows 10 Home Edition 64-bit operating system, and Matlab 2023a as the numerical simulation software platform.

- Metrics

To quantitatively assess the performance of the algorithm, we evaluate the algorithm using two performance metrics, Average Distance Error and its 99% Error Bound. Average Distance Error is a measure of the average distance error between the estimated target position and the true target position. It gives a visual indication of the accuracy of the estimated position, with smaller errors indicating more accurate estimates.

(24) $$error_{ADE}=\frac{1}{N}\sum_{i=1}^{N}∥P_{ture}^{i}-P_{estimate}^{i}∥.$$

$P_{ture}^{i}$ and $P_{estimate}^{i}$ are the true and estimated positions of the *i*-th time, respectively, and *N* is the number of measurements.

99% Error Bounds is based on the concept of confidence intervals; for 99% error bounds, we can use the following formula to estimate:

(25) $$error_{EB}=t_{\beta /2,N-1}\times \frac{s}{\sqrt{N}}.$$

$t_{\beta /2,N-1}$ is the critical value of the t-distribution, corresponding to the confidence level (99%) and the degrees of freedom (N−1), *s* is the standard deviation, and *N* is the sample size.

- Baselines

The baseline methods compared in the numerical simulation experiments are Bearing-Only and Bearing–Angle. Bearing-Only estimates the target state by calculating bearing vectors through the pixel coordinates of the center of the target detection bounding box combined with the pinhole camera model. It is sensitive to changes in target size and requires the observer to perform lateral movements to enhance observability, conflicting with the mission requirements of unmanned aerial vehicles (UAVs). Bearing–Angle, by fusing bearing vectors with the angular span of the detection bounding box, utilizes dimensional information to improve observability and supports direct target-approaching trajectory planning. It employs PLKF to simplify calculations but relies on the accuracy of bounding boxes and is sensitive to noise. In the experiments, public codes of both methods and parameter settings optimized for this task were used for fair comparison to validate the effectiveness of the Dynamic Bearing–Angle.

### 4.2. Numerical Simulation Experimental Scenario Setting

We follow the numerical simulation data generated in the literature [5] for three scenarios to evaluate the performance of the proposed Dynamic Bearing–Angle algorithm.

The initial covariance matrix of the estimated state is set to $P(t_{0})=0.1I$. The target size is a circle with diameter ℓ=1. The target size is a circle with diameter B. The target size is a circle with diameter B. The update rate of the system is 50 Hz.

We use the same parameter values in all simulation examples to verify the robustness of the algorithm. Better performance can be achieved if the parameters are well tuned for a specific scenario. We perform an $N_{x}=100$ Monte Carlo simulation for each scenario.

- Numerical Simulation Scenario 1:

The target is at rest, and the observer moves in a circle around the target. At this point, the target is located at $P_{T}={\left[0,10\right]}^{T}$, and the observer is moving at a speed of 3 m/s around a circle with a radius of 5 m. The initial estimated state of the system is ${\hat{P}}_{O}\left(t_{0}\right)={\left[0,13\right]}^{T}$, ${\hat{v}}_{O}\left(t_{0}\right)={\left[0,0\right]}^{T}$, ${\hat{\ell }}_{O}\left(t_{0}\right)=1.6$.

- Numerical Simulation Scenario 2:

The target is at rest, and the observer moves closer to and further away from the target in a straight line. At this point, the target is at ${\hat{P}}_{O}\left(t_{0}\right)={\left[0,10\right]}^{T}$, and the observer moves back and forth towards the target in a straight line with constant acceleration −2 m/$s^{2}$.

The initial conditions are $P_{O}\left(t_{0}\right)={\left[0,5\right]}^{T}$, $v_{O}\left(t_{0}\right)={\left[0,4\right]}^{T}$.

The initial estimated state of the system is ${\hat{P}}_{O}\left(t_{0}\right)={\left[0,13\right]}^{T}$, ${\hat{v}}_{O}\left(t_{0}\right)={\left[0,0\right]}^{T}$, ${\hat{\ell }}_{O}\left(t_{0}\right)=0.8$.

- Numerical Simulation Scenario 3:

The target moves at a constant velocity, and the observer is controlled by proportional navigation guidance (PNG) laws to approach the target. In the process, both orientation and angle are changed. For the target velocity, the observer velocity $v_{T}={\left[\frac{1}{\sqrt{2}},\frac{1}{\sqrt{2}}\right]}^{2}$ magnitude is constant at 3 m/s, and the velocity direction is controlled by the PNG law [30]. The navigation gain of the PNG law is selected as 1. The initial estimation of the target state is the same as in Scene 1.

### 4.3. Comparison with the State of the Arts

In this section, we evaluate three algorithms, Bearing-Only [4], Bearing–Angle [5], and Dynamic Bearing–Angle; we set up three different motion scenarios, and in the same scenarios, we set up randomly fluctuating measurement noises with standard deviations of 0.01, 0.015, and 0.03, respectively. The average distance error of each algorithm was calculated and compared under each noise condition. It should be noted that these three orders of magnitude of noise used for testing were determined by the maximum noise conditions encountered in actual testing (which can be derived from the values of the center offset of the detection frame shown in Figure 1b combined with Equations (3) and (4)).

**Figure 5:**
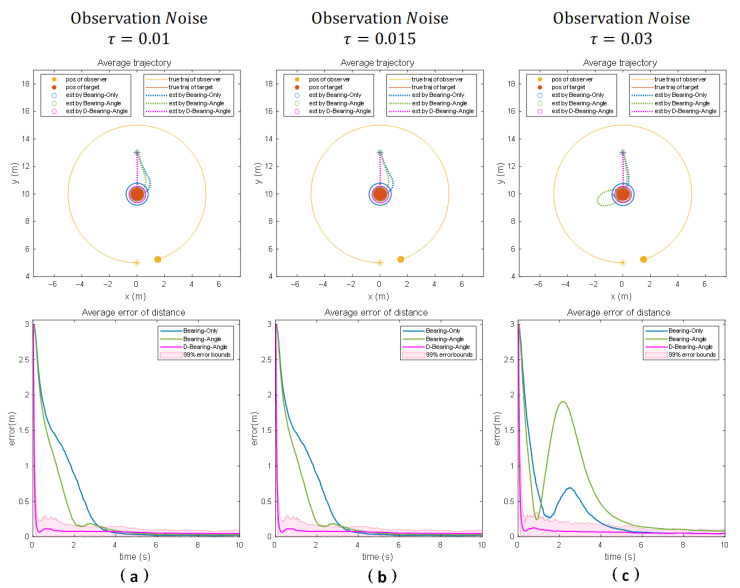
The motion scenarios are the results of numerical simulations for 100 Monte Carlo runs for three different noise scenarios when the observer moves in a circle around the target (Columns (**a**–**c**) show the experimental results for different noise intensities, respectively). The “*” denotes the initial position, the solid line denotes the true trajectory, the dashed line denotes the estimated trajectory, the solid circle denotes the observer and the target, and the hollow circle denotes the estimated target position. The second row of the figure shows the average distance error, which is used to measure the estimation error of the distance between the observer and the target.

- Results under scenario 1:

Figure 5a demonstrates the estimation knots of each algorithm under motion scenario 1 and that the standard deviation of observation noise is 0.01. At this time, the observation noise is small, the observation error of the system has a relatively limited impact on the performance of the algorithms, and the average distance errors corresponding to the three algorithms are kept small. From the figure, we can observe that the Dynamic Bearing–Angle response is the fastest, which may be the case when the noise is small or the system state changes slowly, and the dynamic tuning may allow the filter to obtain better estimation results without a large number of iterations.

Figure 5b shows the estimation results of the different algorithms when the standard deviation of the noise increases to 0.015. The effect of observation noise on the performance of the algorithm gradually increases, and the stability of the system localization decreases. Bearing-Only is affected by only a single measurement noise in the simpler motion scenarios because it only relies on the target’s azimuthal angle information; thus, its stability decreases, but it can still maintain convergence after a period of adjustment. On the other hand, Bearing–Angle needs to combine the target’s azimuth and angle information, so its stability is greatly affected by the noise, which generates large measurement fluctuations, and although the final localization accuracy error can be converged to a smaller value, its stability is poorer. In contrast, the Dynamic Bearing–Angle algorithm can still deal with the interference caused by the noise better, showing excellent robustness, significantly better than the other two algorithms. However, due to its dynamic adjustment, the final localization accuracy error is slightly worse than that of Bearing-Only and Bearing–Angle.

When the noise standard deviation further increases to 0.03 (as shown in Figure 5c), the observation noise of the system has a significant impact on the algorithm performance. At this time, both Bearing-Only and Bearing–Angle fail to converge; the Dynamic Bearing–Angle response speed and localization accuracy are also reduced, but the algorithm still maintains its robustness and significantly outperforms the other two algorithms.

**Figure 6:**
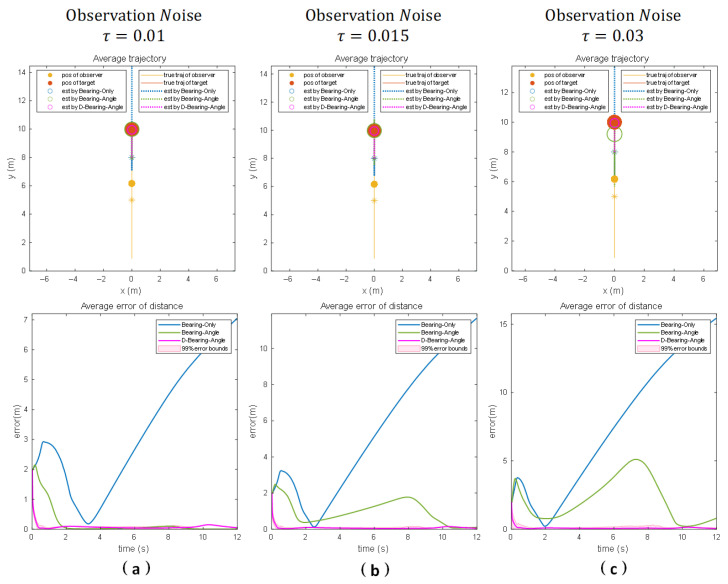
Numerical simulation results for 100 Monte Carlo runs for three different noise scenarios when the observer makes reciprocal linear motion towards the target for the motion scenario. (Columns (**a**–**c**) show the experimental results for different noise intensities, respectively). The “*” denotes the initial position, the solid line denotes the true trajectory, the dashed line denotes the estimated trajectory, the solid circle denotes the observer and the target, and the hollow circle denotes the estimated target position.

- Results under scenario 2:

Figure 6a shows the estimation results of each algorithm under motion scenario 2 and with an observation noise standard deviation of 0.01. It can be seen that Bearing-Only diverges because the system motion state does not have the corresponding lateral acceleration, and Bearing–Angle and Dynamic Bearing–Angle converge and are able to locate the target position more accurately, which proves that these two algorithms have strong observability. We also note that Dynamic Bearing–Angle still has a faster response than the other algorithms.

When we increase the standard deviation of the observation noise to 0.015 and 0.03 (as shown in Figure 6b and Figure 6c, respectively), then Bearing-Only diverges and Bearing–Angle and Dynamic Bearing–Angle converge. However, as the noise increases, Bearing–Angle fluctuates and convergence slows down due to the change in the motion state of the system. In contrast, Dynamic Bearing–Angle still maintains better response speed, stability, and localization accuracy, which fully demonstrates the superiority of the algorithm proposed in this paper.

**Figure 7:**
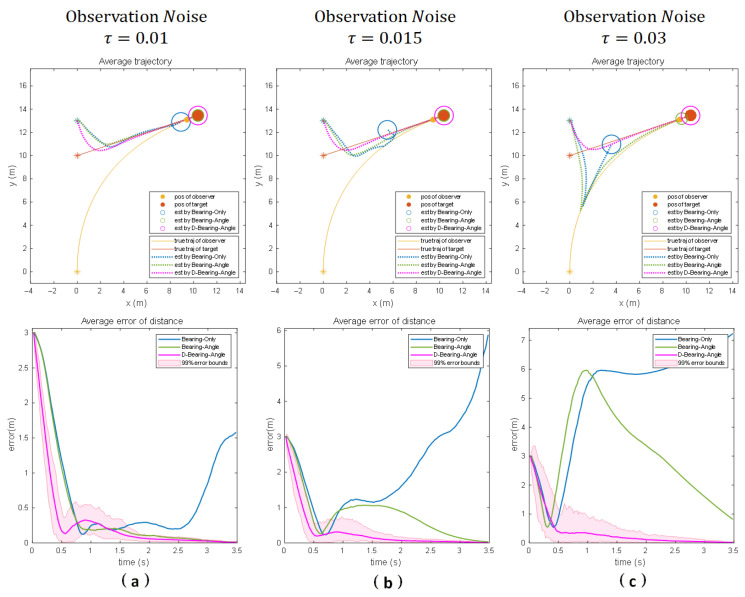
Numerical simulation results for 100 Monte Carlo runs for three different noise scenarios when the motion scenario is a target moving at a constant velocity and the observer is controlled by the proportional navigation guidance (PNG) law to approach the target. (Columns (**a**–**c**) show the experimental results for different noise intensities, respectively). The “*” denotes the initial position, the solid line denotes the true trajectory, the dashed line denotes the estimated trajectory, the solid circle denotes the observer and the target, and the hollow circle denotes the estimated target position.

- Results under scenario 3:

Figure 7a illustrates the estimation results of each algorithm in Scene 3 in motion and with a standard deviation of 0.01 in the observation noise. It can be seen that both Bearing–Angle and Dynamic Bearing–Angle successfully converge before the collision occurs. Due to the small lateral motion of the observer and its weak observability, Bearing-Only is completely unable to estimate the state of the target. When we increase the observation noise to 0.015 and 0.03 (as shown in Figure 7b and Figure 7c, respectively), Bearing–Angle is affected by the larger noise, and although there is a tendency to converge eventually, the localization accuracy fluctuates to a greater extent with the noise increase in the process. In contrast, the Dynamic Bearing–Angle algorithm proposed in this paper always maintains high robustness and localization accuracy and is less affected by noise.

### 4.4. Comparison with Other Adaptive Filtering Algorithms

To further validate the robustness of the proposed algorithm in dynamic noise environments, this section benchmarks the Dynamic Bearing–Angle framework against four classical adaptive filters: Sage–Husa [25], Adaptive Unscented Kalman Filter (AUKF) [27], Adaptive Particle Filter (APF) [26], and Adaptive Extended Kalman Filter (AEKF) [29]. Experiments are conducted under the three typical scenarios described in Section 4.2, with three levels of measurement noise intensity applied to each scenario.

**Figure 8:**
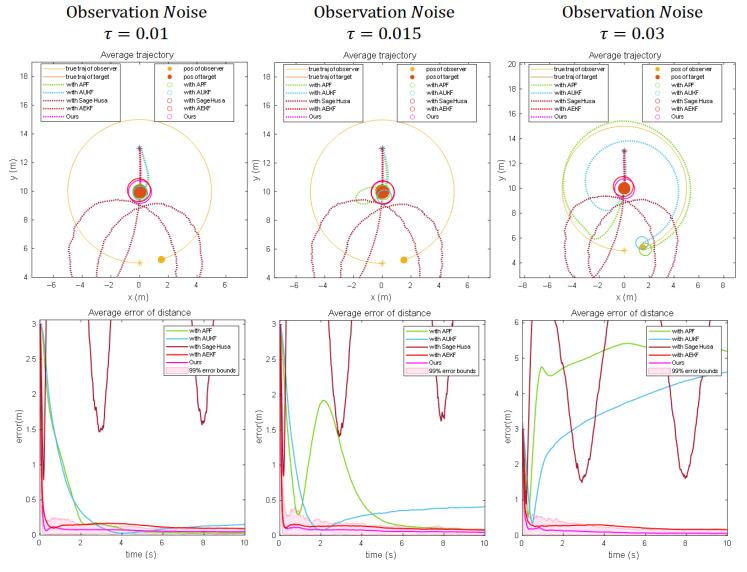
Results using the Dynamic Bearing–Angle algorithmic framework with different optimization algorithms for scenario 1. The “*” denotes the initial position, the solid line denotes the true trajectory, the dashed line denotes the estimated trajectory, the solid circle denotes the observer and the target, and the hollow circle denotes the estimated target position.

**Figure 9:**
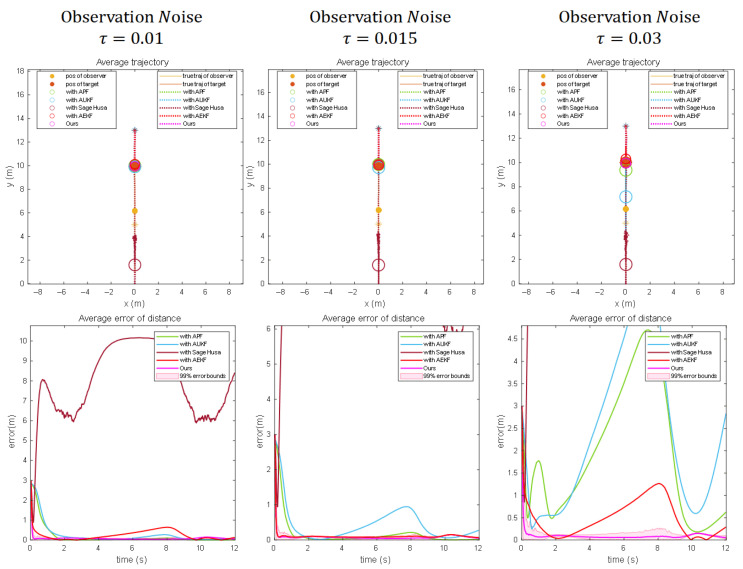
Results using the Dynamic Bearing–Angle algorithmic framework with different optimization algorithms for scenario 2. The “*” denotes the initial position, the solid line denotes the true trajectory, the dashed line denotes the estimated trajectory, the solid circle denotes the observer and the target, and the hollow circle denotes the estimated target position.

**Figure 10:**
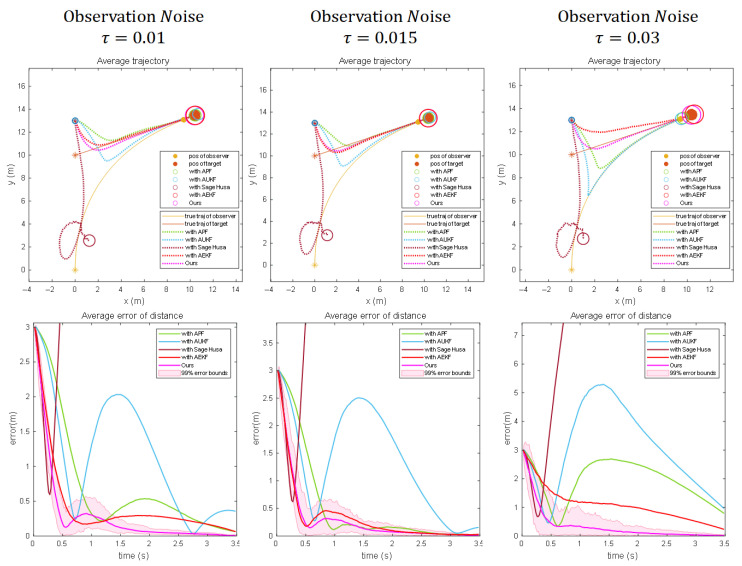
Results using the Dynamic Bearing–Angle algorithmic framework with different optimization algorithms for scenario 3. The “*” denotes the initial position, the solid line denotes the true trajectory, the dashed line denotes the estimated trajectory, the solid circle denotes the observer and the target, and the hollow circle denotes the estimated target position.

Figure 8, Figure 9 and Figure 10 evaluate the effectiveness of the proposed optimizer compared to other adaptive filtering algorithms. When integrating the Sage–Husa adaptive filter into the Dynamic Bearing–Angle framework, the algorithm exhibits rapid convergence but also susceptibility to divergence. This behavior originates from its design logic of dynamically adjusting noise parameters. During the initial phase, real-time updates to statistical estimates of system and measurement noise enable swift adaptation to environmental changes, resulting in fast convergence. However, this adaptability introduces vulnerability: when external noise abruptly exceeds the algorithm’s predefined adjustment range or accumulates beyond a threshold, continuous corrections to noise parameters gradually deviate from ground truth. Such deviations form a positive feedback loop through the coupling of state estimation and noise adjustment, sharply increasing error sensitivity and ultimately triggering exponential divergence in estimation results.

When employing AUKF as the optimizer, the algorithm exhibits fluctuating precision, a consequence of inherent contradictions in its hybrid architecture. While deterministic sampling strategies capture state distributions in nonlinear systems more efficiently than stochastic methods, the introduced noise parameter adaptation mechanism disrupts the stable propagation process. In dynamically changing environments, periodic mismatches arise between the adjustment step size of noise estimation and the deterministic state propagation path. These mismatches force the algorithm to oscillate between “tracking current observations” and “maintaining historical trajectories,” manifesting as time-varying precision fluctuations akin to a damped system oscillating near equilibrium.

The APF optimizer demonstrates high precision but slow convergence, reflecting fundamental principles of probabilistic sampling. By deploying a large particle swarm to exhaustively search the state space, followed by iterative weight filtering and resampling to focus on high-probability regions, the algorithm accurately characterizes complex non-Gaussian distributions. However, the computational burden of propagating states, calculating weights, and reconstructing distributions for all particles in each iteration is substantial. Moreover, particle swarm optimization is inherently gradual—initial phases require sufficient time for low-weight particles to be eliminated and high-weight particles to cluster near the true state, inherently limiting convergence speed.

The experiments further evaluate the performance of the Adaptive Extended Kalman Filter (AEKF) in dynamic scenarios. AEKF demonstrates advantages in noise adaptability: by updating the measurement noise covariance matrix in real time, it effectively suppresses medium-intensity noise interference in state estimation, achieving positioning errors under stable observation conditions that are second only to the proposed Dynamic Extended Kalman Filter (DEKF). However, the critical distinction between AEKF and DEKF lies in the latter’s adaptive smoothing factor adjustment mechanism—this mechanism dynamically regulates the update intensity of the process noise covariance matrix through real-time analysis of residual sequence statistical characteristics. Specifically, when abrupt changes in system dynamics or anomalies in noise statistics are detected, DEKF autonomously enhances the smoothing factor to mitigate overfitting risks while reducing the factor under stable conditions to accelerate parameter convergence. In contrast, AEKF’s fixed smoothing factor design introduces two inherent limitations: (1) the static process noise covariance combined with the fixed smoothing factor restricts rapid response capability during sudden target motion pattern transitions, resulting in delayed correction phenomena; (2) in non-Gaussian noise environments, abrupt shifts in noise statistics—due to the absence of dynamic smoothing factor compensation—trigger error accumulation. This forces AEKF to alleviate model mismatch solely by increasing the Kalman gain, yet this strategy lacks dynamic decoupling capability for process noise, ultimately leading to significant error amplification and violent fluctuations in error curves.

## 5. Real-World Experimental Results

Experiments based on the MAV6D dataset, containing multi-condition UAV flight data, are conducted. Quantitative analysis and qualitative visualization validate the algorithm’s robustness in low/high-speed scenarios. Ablation experiments quantify the contribution of each module, confirming the synergistic advantages of the dual mechanism.

### 5.1. Experimental Dataset

In the experimental validation with real sensor data, we leverage the image sequences and annotation information of the MAV6D dataset [31] to verify the motion estimation algorithm. This dataset consists of image data of unmanned aerial vehicles (UAVs) under various flight conditions, including the ground-truth position information corresponding to each image and the intrinsic/extrinsic parameters of the monocular camera. The MAV6D dataset is obtained through an indoor automatic data collection method using a precise acquisition platform comprising a VICON motion capture system, a monocular camera, and a UAV. Specifically, the VICON system is mounted around the indoor space, taking the world coordinate system as the benchmark, and captures markers synchronously via multiple cameras to provide high-precision 6D pose ground-truth of the UAV at a sampling frequency of 100 Hz. The monocular camera is fixed at an indoor perspective facing the flight area, and its image data with a resolution of 640 × 480 pixels and a sampling frequency of 20 Hz needs to be calibrated to determine the transformation relationship with the world coordinate system. The target UAV flies within an indoor range of 1–6 m, with nine keypoints (one centroid point + eight corner points) defined on its body and VICON reflective markers attached, and the three components are synchronized by timestamps to ensure data consistency. Containing 57,075 annotated images divided into 129 video segments, the dataset covers various flight conditions, such as different UAV maneuvering states, perspective changes (e.g., top-down/side views), and distance variations (1–6 m) between the MAV and the camera. The controlled indoor lighting (without direct strong light or dynamic shadows, with data augmentation simulating lighting changes) and static backgrounds (e.g., laboratory walls) reduce occlusion interference. This dataset is designed to evaluate the estimation accuracy and robustness of the algorithm for UAV position changes in real-world scenarios, laying a solid foundation for the algorithm’s practical applications.

### 5.2. Test Results with Actual Sensor Data

#### 5.2.1. Quantitative Analysis Results

We conducted a comprehensive test on the MAV6D dataset with four typical frame intervals (0–20, 21–40, 41–100, and after 100 frames) and evaluated the target position estimation accuracy using Mean Error (ME) and Root Mean Square Error (RMSE). The results are shown in Table 1.

The experimental results show that the Dynamic Bearing–Angle algorithm significantly outperforms the Bearing-Only and Baseline–Angle methods in estimating the target position in all frame intervals in the comparative tests on the MAV6D dataset. In the initial stage, the algorithm has a fast response speed and good adaptability to noise, and it can stabilize the estimation error faster than the traditional methods; in the high-speed maneuvering scenario, the dual-robust mechanism effectively deals with the dynamic noise such as the detection frame jitter and continuously maintains the error convergence; in the process of long-time tracking, the algorithm maintains a low level of error by dynamically adjusting the noise covariance and the suppression of the outliers, while the other methods show obvious fluctuation of the error with the passage of time. The experimental results fully verify the strong robustness of the algorithm to noise in real complex scenes and the stability of long-term estimation.

#### 5.2.2. Results of Qualitative Analysis of Examples

In order to more clearly compare the specific differences between the methods, we visualize two typical examples in detail. In the context of target detection and tracking, the patterns of bounding box noise interference are closely related to the target’s motion speed, which can be divided into two typical scenarios for target motion estimation: small-amplitude bias noise interference under low-speed motion and large-amplitude deviation noise interference under high-speed motion.

- Small amplitude deviation noise at low-speed motion:

When the target is in a low-speed motion state, the detection bounding box is primarily affected by stationary observation noise. This type of noise originates from inherent sensor errors or slow time-varying environmental disturbances, with statistical characteristics approximating stationarity and an error distribution following a zero-mean Gaussian distribution. The standard deviation is typically at the pixel-level or centimeter-level. Under the influence of this noise, although the position of the detection bounding box globally converges to the ground truth, random offsets independent of the target velocity exist.

**Figure 11:**
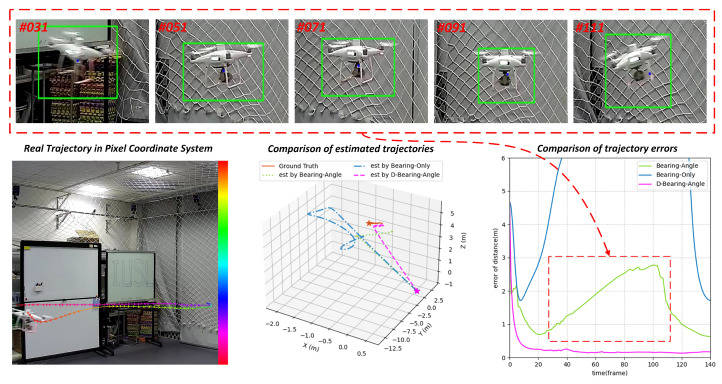
Results of experiments in low-speed motion scenarios of UAV. Dynamic Bearing–Angle possesses high accuracy and strong robustness, but Bearing-Only and Bearing–Angle work unstably.

As shown in the visualization results of Figure 11, in the low-speed motion scenario of the drone, Bearing-Only fails to converge due to insufficient state observability caused by the lack of lateral acceleration modeling. While Bearing–Angle converges rapidly in the early stage, the PLKF (Pseudo-Linear Kalman Filter) it employs introduces approximation errors due to the truncation of high-order terms during pseudo-linearization. When the bounding box jitter exceeds the critical threshold, accumulated errors can cause systematic deviations in the estimated trajectory. Even small random offsets in the detection bounding box can lead to a decline in the robustness and estimation accuracy of the Bearing–Angle algorithm due to long-term noise accumulation. In contrast, the Dynamic Bearing–Angle algorithm significantly enhances adaptability to random noise fluctuations by introducing an adaptive dynamic Kalman filtering mechanism, which effectively tracks time-varying noise characteristics.

- Large deviation noise at high-speed motion:

When the target performs high-speed maneuvers, the detection bounding box faces coupled interference from non-stationary dynamic noise and observation lag errors. On one hand, the traditional constant-velocity motion model fails to characterize sudden changes in target acceleration, leading to non-linear growth of state prediction errors as velocity increases. On the other hand, issues such as image blur and insufficient sensor frame rate caused by high-speed motion introduce impulsive observation outliers. This type of noise exhibits strong time-varying characteristics, with error distributions showing heavy-tailed properties, potentially causing the detection bounding box to deviate from the ground truth by more than 50% of the target size, or even leading to tracking loss.

**Figure 12:**
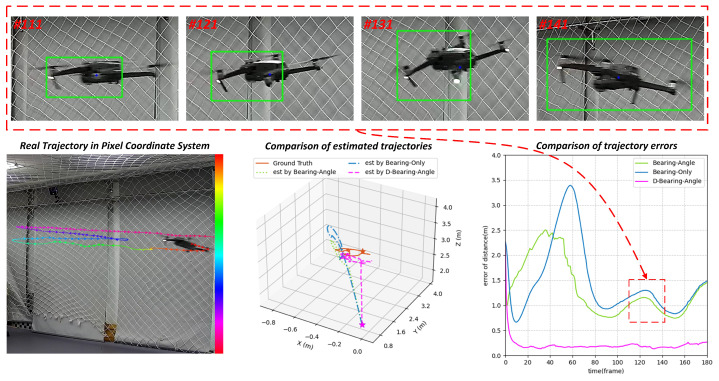
Results of experiments in UAV fast maneuvering scenarios. Dynamic Bearing–Angle possesses high accuracy and strong robustness, but Bearing-Only and Bearing–Angle work unstably.

As shown in the visualization results of Figure 12, in high-speed motion scenarios, the detection bounding box is subjected to impulsive observation outliers, causing fluctuations in the estimation accuracy of Bearing-Only and Bearing–Angle, which reflects the poor robustness of these algorithms. In contrast, Dynamic Bearing–Angle performs well, with the predicted trajectory closely matching the ground truth trajectory. The error curve demonstrates that the algorithm maintains good robustness and precision throughout the entire image sequence. This is attributed to the fact that the proposed algorithm not only dynamically adapts to Gaussian noise that changes with the motion state of the detection bounding box but also introduces M-estimation to handle extreme outliers, enabling the algorithm to maintain high-precision estimation while possessing strong robustness.

### 5.3. Ablation Experiment

- Results of ablation experiments on the MAV6D dataset:

To quantify the contribution of M-estimation and dynamic smoothing factor to the performance of the algorithm, four sets of controlled experiments are designed on the MAV6D dataset: the traditional Bearing–Angle algorithm (Baseline) based on AEKF filtering without introducing any robust mechanism, Baseline+M- estimation, Baseline+Dynamic smoothing factor with only adaptive α to dynamically update the noise covariance, and Dynamic Bearing–Angle, a complete scheme that incorporates dual robustness mechanisms, are tested on four typical frame intervals (0–20, 21–40, 41–100, and after 100 frames).

The results are shown in Table 2. The experimental results show that the Dynamic Bearing–Angle algorithm significantly outperforms the control methods in overall performance. Its Mean Error (ME) and Root Mean Square Error (RMSE) remain the lowest across all frame intervals (0–20, 21–40, 41–100, and after 100 frames). The algorithm demonstrates faster convergence in the initial stage, a further substantial error reduction during mid-term high-speed maneuvers, and outstanding long-term stability with errors converging to approximately 1/6 of the baseline method.

In the ablation analysis of modules, M-estimation effectively suppresses sudden detection frame noise but fails to adapt to time-varying noise characteristics without dynamic covariance adjustment, as evidenced by error rebound in the later stage. The dynamic smoothing factor accelerates adaptive updates of noise covariance but shows insufficient suppression of abrupt outliers. The combined dual mechanism reduces mid-term errors by over 50% compared to single modules, forming a cascade effect of “outlier suppression followed by covariance adaptation” through cross-level complementarity, which significantly enhances the algorithm’s robustness and accuracy in dynamic noise environments.

**Figure 13:**
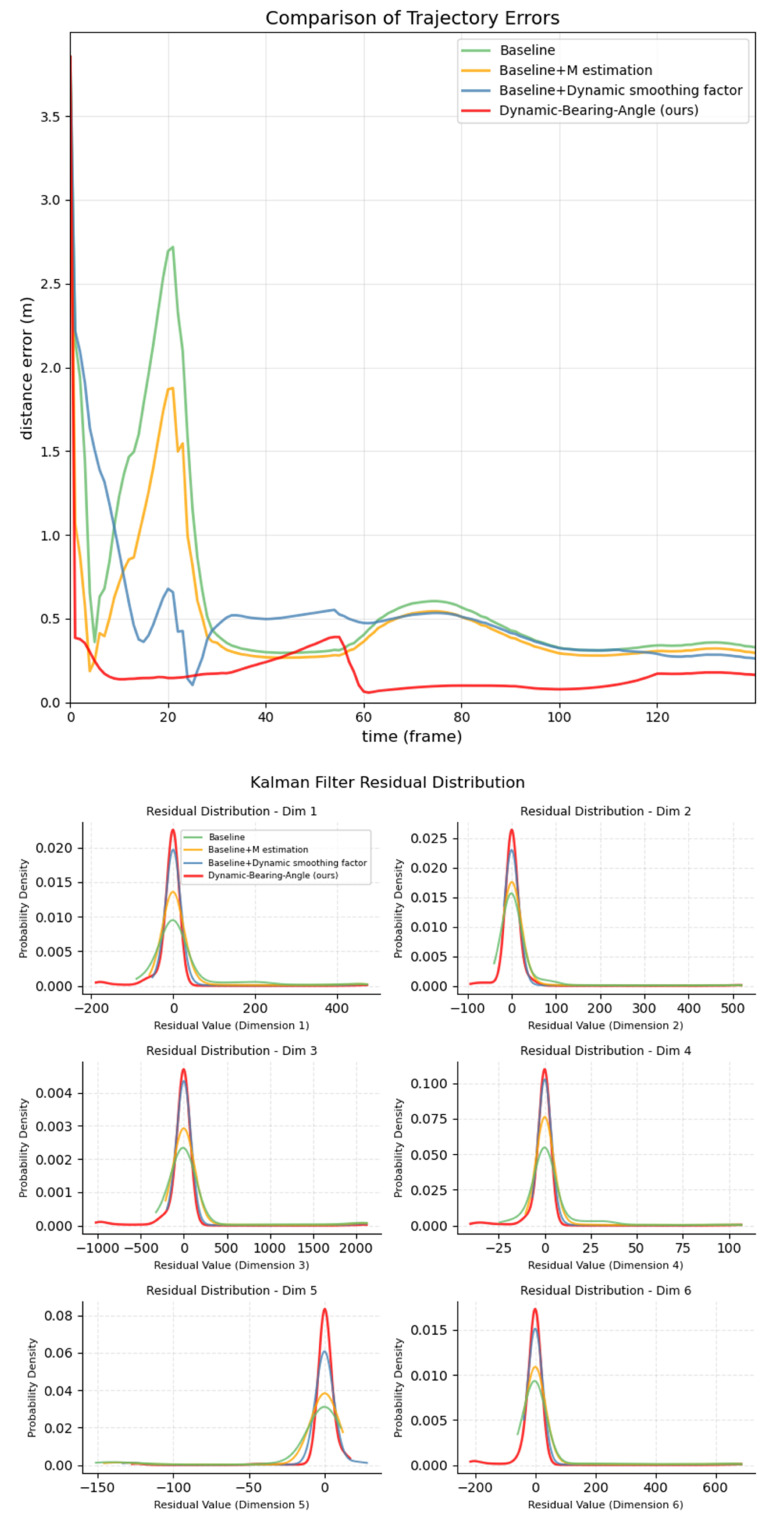
Comparison of distance error curve and residual probability density function for an instance.

- Validation of validity using probability density functions of residuals:

To further elucidate the capability of each module in handling estimation errors, we compared the residual probability density functions generated during the filtering process of four methods in one case, with the results presented in Figure 13.

An ideal residual distribution should exhibit a zero mean, narrow variance, and symmetric Gaussian shape, which correspond to unbiasedness, high precision, and robustness, respectively. The following analysis is conducted from the perspective of method comparison:

The multidimensional residuals of the baseline method exhibit obvious issues: they not only have mean shifts (e.g., a right shift in Dim1 and a left shift in Dim3) but also feature a wide distribution range (with tails extending beyond ±100) and unilateral long-tail characteristics (which are directly associated with sudden disturbances or model mutations). Although adding M-estimation or dynamic smoothing alone can alleviate biases and reduce tail extent to a certain degree, they have significant limitations: the former fails to handle the dynamic changes of noise, while the latter struggles to adapt to the periodicity and variability of azimuth angles, resulting in limited improvement. In contrast, the complete DBA method, through the synergy of dual robust mechanisms, achieves strict alignment of residual peaks with the zero mean across all dimensions, forming a sharp and symmetric unimodal distribution. Its full-process collaborative optimization from model adaptation and noise tracking to outlier suppression makes the residual distribution closer to the ideal Gaussian shape.

## 6. Conclusions

The core contribution of this paper is to propose a novel Dynamic Bearing–Angle visual target motion estimation algorithm, which effectively solves the limitations of traditional methods in complex dynamic environments by fusing M-estimation and dynamic filtering techniques. Starting from analyzing the shortcomings of existing visual target motion estimation algorithms, the research work carefully designs the Dynamic Bearing–Angle algorithm to address its high dependence on the detection frame accuracy and its performance degradation in non-Gaussian noise environments. The dual robust mechanism of this algorithm enables it to maintain high accuracy and stability in the face of rapid target maneuvering and detection noise interference. Through a series of rigorous numerical simulations and real data tests, the superior performance of the algorithm is verified in a variety of complex scenarios. This result not only makes an important contribution to the field of visual target motion estimation theoretically but also has a wide range of applications in practical applications, such as UAV formation, anti-UAV detection, and UAV tracking missions, which are expected to significantly improve the performance and reliability of related systems.

## Figures and Tables

**Table 1:** Performance evaluation of contrasting methods on the MAV6D dataset.

Method	Frame 0 to 20		Frame 21 to 40		Frame 41 to 100		Frame After 100		Average FPS
	ME	RMSE	ME	RMSE	ME	RMSE	ME	RMSE	
Bearing-Only	2.7629	3.6741	2.8384	3.2344	1.9722	2.0180	2.4395	2.8725	6667.08
Baseline–Angle	2.1502	2.2752	1.4824	1.4825	1.1017	1.1349	1.3070	1.5678	2940.63
Dynamic Bearing–Angle	1.2404	1.5808	0.4986	0.7352	0.4237	0.4814	0.3631	0.3660	2142.72

**Table 2:** Ablation study: performance of core modules.

Method	Frame 0 to 20		Frame 21 to 40		Frame 41 to 100		Frame After 100	
	ME	RMSE	ME	RMSE	ME	RMSE	ME	RMSE
Baseline	2.1922	2.3080	1.4927	1.9105	1.4123	1.4124	1.2103	1.2199
Baseline + M-estimation	1.4921	1.5385	1.0055	1.0177	0.8645	0.9211	1.0421	1.1699
Baseline + Dynamic smoothing factor	1.4714	1.5202	0.8427	0.8642	0.8859	0.9359	1.0001	1.0858
Dynamic Bearing–Angle	1.2404	1.5808	0.4986	0.7352	0.4237	0.4814	0.3631	0.3660

## Data Availability

The datasets generated and analyzed during the current study are not publicly available due Naval University of Engineering requirements but are available from the corresponding author on reasonable request.

## References

[1] Minaeian S., Liu J., Son Y.J. (2015). Vision-based target detection and localization via a team of cooperative UAV and UGVs. IEEE Trans. Syst. Man Cybern. Syst..

[2] Wang B., Li Q., Mao Q., Wang J., Chen C.P., Shangguan A., Zhang H. (2024). A Survey on Vision-Based Anti Unmanned Aerial Vehicles Methods. Drones.

[3] Yang K., Bai C., She Z., Quan Q. (2024). High-Speed Interception Multicopter Control by Image-based Visual Servoing. arXiv.

[4] Sui D., Deghat M., Sun Z., Greiff M. (2024). Unbiased bearing-only localization and circumnavigation of a constant velocity target. IEEE Trans. Intell. Veh..

[5] Ning Z., Zhang Y., Li J., Chen Z., Zhao S. (2024). A bearing-angle approach for unknown target motion analysis based on visual measurements. Int. J. Robot. Res..

[6] Khather S.I., Ibrahim M.A., Ibrahim M.H. (2024). PID Controller for A Bearing Angle Control in Self-Driving Vehicles. J. Robot. Control (JRC).

[7] Hoelzer H., Johnson G., Cohen A. (1978). Modified polar coordinates-the key to well behaved bearings only ranging. IR D Rep..

[8] Fogel E., Gavish M. (1988). Nth-order dynamics target observability from angle measurements. IEEE Trans. Aerosp. Electron. Syst..

[9] He S., Shin H.S., Tsourdos A. (2019). Trajectory optimization for target localization with bearing-only measurement. IEEE Trans. Robot..

[10] Li J., Ning Z., He S., Lee C.H., Zhao S. (2022). Three-dimensional bearing-only target following via observability-enhanced helical guidance. IEEE Trans. Robot..

[11] Aidala V.J., Nardone S.C. (1982). Biased estimation properties of the pseudolinear tracking filter. IEEE Trans. Aerosp. Electron. Syst..

[12] Hammel S.E., Liu P.T., Hilliard E.J., Gong K.F. (1989). Optimal observer motion for localization with bearing measurements. Comput. Math. Appl..

[13] Sabet M., Fathi A., Daniali H.M. (2016). Optimal design of the own ship maneuver in the bearing-only target motion analysis problem using a heuristically supervised extended Kalman filter. Ocean Eng..

[14] Anjaly P., Ratnoo A. (2018). Observability enhancement of maneuvering target with bearings-only information. J. Guid. Control Dyn..

[15] Yang Y., Liu Z., Qin Y., Pan Q. (2023). Novel pseudo-linear Kalman filtering for 3D angle-only tracking in the presence of observer’s location errors. Automatica.

[16] Asgharian M. (2014). On the singularities of the information matrix and multipath change-point problems. Theory Probab. Its Appl..

[17] Griewank A. (1985). On solving nonlinear equations with simple singularities or nearly singular solutions. SIAM Rev..

[18] Zhang Y., Guo Z., Wu J., Tian Y., Tang H., Guo X. (2022). Real-time vehicle detection based on improved yolo v5. Sustainability.

[19] Aidala V.J. (1979). Kalman filter behavior in bearings-only tracking applications. IEEE Trans. Aerosp. Electron. Syst..

[20] Lin X., Kirubarajan T., Bar-Shalom Y., Maskell S. Comparison of EKF, pseudomeasurement, and particle filters for a bearing-only target tracking problem. Proceedings of the Signal and Data Processing of Small Targets 2002.

[21] Griffin B.A., Corso J.J. Depth from camera motion and object detection. Proceedings of the IEEE/CVF Conference on Computer Vision and Pattern Recognition.

[22] Rodriguez A., Moreno F. (2015). Evolutionary computing and particle filtering: A hardware-based motion estimation system. IEEE Trans. Comput..

[23] Nardone S.C., Aidala V.J. (1981). Observability criteria for bearings-only target motion analysis. IEEE Trans. Aerosp. Electron. Syst..

[24] Lingren A.G., Gong K.F. (1978). Position and velocity estimation via bearing observations. IEEE Trans. Aerosp. Electron. Syst..

[25] Narasimhappa M., Mahindrakar A.D., Guizilini V.C., Terra M.H., Sabat S.L. (2019). MEMS-based IMU drift minimization: Sage Husa adaptive robust Kalman filtering. IEEE Sens. J..

[26] Fox D. (2001). KLD-sampling: Adaptive particle filters. Advances in Neural Information Processing Systems 14 (NIPS).

[27] Sun F., Hu X., Zou Y., Li S. (2011). Adaptive unscented Kalman filtering for state of charge estimation of a lithium-ion battery for electric vehicles. Energy.

[28] Mohamed A., Schwarz K. (1999). Adaptive Kalman filtering for INS/GPS. J. Geod..

[29] Akhlaghi S., Zhou N., Huang Z. Adaptive adjustment of noise covariance in Kalman filter for dynamic state estimation. Proceedings of the 2017 IEEE Power & Energy Society General Meeting.

[30] Cho N., Kim Y. (2016). Modified pure proportional navigation guidance law for impact time control. J. Guid. Control Dyn..

[31] Zheng Y., Zheng C., Shen J., Liu P., Zhao S. (2024). Keypoint-guided efficient pose estimation and domain adaptation for micro aerial vehicles. IEEE Trans. Robot..

